# A New Remedy in Gonorrhœa

**Published:** 1866-08

**Authors:** J. S. Prettyman

**Affiliations:** Milford, Delaware


*A New Remedy in Gonorrhœa.* By J. S. Prettyman, M. D., of Milford, Delaware.

In July, 1859, while narrowly observing the effects of oil of erigeron administered in a fearful hæmoptysis, I was led to suspect that it would prove a useful remedy in the treatment of gonorrhœa. Acting upon this presumption, I immediately commenced giving it to a patient then under my care, in whose case all the vaunted specifics had most signally failed. He improved at once, and was speedily cured. Since that date I have prescribed it in about fifty cases, with unvarying success. It arrests the discharge in about seventy-two hours, and effects a cure in from six to eight days. I do not recommend it as a specific in all cases, but design merely to bring it to the notice of the profession as an exceedingly valuable medicine in this disease. Of course all scientific medical practice is based upon the well-known pathological condition of the structures involved, and this is our unerring guide. When, in recent cases, the urethral inflammation is severe, my plan is to precede the remedy with a full dose of some active hydragogue. A good formula is: ℞.—Pulv. senna ℈ij; pulv. jalapa ℈j; pulv. aromaticus gr. x. M. Add a gill of boiling water and a teaspoonful of sugar, and, when sufficiently cool, agitate, and swallow at a dose. As soon as this operates, give ten drops of the oil on sugar, and three hours later a full dose of spts. æther. nit. in infus. althea, and so on every three hours alternately until the urethral irritation is allayed. Then leave off the latter, and continue the oil until the cure is complete. If the case is not recent, or there is but little urethral irritation, the oil alone is sufficient.

I have used it also in combination with copaiba and other articles, and found such preparations to answer a good purpose, but no better than the oil alone.

The oil which I use is reputed to be that of the *Erigeron Canadense;* but I presume that from the *Philadelphicum* is equal, if not superior, for this purpose.—*American Journal of the Medical Sciences.*

